# SirT7-mediated transcription of fascin in hyperglycemic glomerular endothelial cells contributes to EndMT in diabetic nephropathy: SirT7-mediated transcription of fascin contributes to EndMT

**DOI:** 10.3724/abbs.2024002

**Published:** 2024-03-07

**Authors:** Mengchen Wu, Yingxiang Hao, Xinwan Wu, Minmin Zhu, Xiangyuan Chen, Jie Qi, Zhuang Yu, Hongjiao Xu

**Affiliations:** 1 Department of Anesthesiology Shanghai General Hospital Shanghai Jiao Tong University School of Medicine Shanghai 201620 China; 2 Department of Anesthesiology Department of Oncology Fudan University Shanghai Cancer Center Shanghai Medical College Fudan University Shanghai 200032 China

**Keywords:** endothelial-to-mesenchymal transition, diabetic nephropathy, glomerular endothelial cell, SirT7

## Abstract

Diabetic nephropathy (DN) is the main cause of end-stage renal disease worldwide. It is reported that the endothelial-to-mesenchymal transition (EndMT) in glomerular endothelial cells plays an important role in DN. As a specific form of epithelial-to-mesenchymal transition, EndMT may involve common regulators of epithelial-to-mesenchymal transition. Fascin has been shown to mediate epithelial-to-mesenchymal transition. In addition, SirT7 has been confir med to contribute to inflammation in hyperglycemic endothelial cells via the modulation of gene transcription. In this study, we speculate that SirT7 modulates fascin transcription and is thus involved in EndMT in hyperglycemic glomerular endothelial cells. Our data indicate that α-smooth muscle actin (α-SMA) and fascin levels are increased, while CD31 levels are decreased in the kidneys of DN rats. Consistently, our cellular experiments reveal that high glucose treatment elevates fascin levels and induces EndMT in human glomerular endothelial cells (HGECs). Moreover, silencing of *fascin* inhibits EndMT in hyperglycaemic HGECs. In addition, SirT7 is found to be decreased in hyperglycemic cells and in the kidneys of DN mice. Moreover, the inhibition of SirT7 increases fascin level and mediates EndMT. An increase in SirtT7 expression decreases fascin expression, inhibits EndMT, and improves renal function in hyperglycemic cells and DN mice. SirT7 is found to bind to the promoter region of *fascin*. In summary, the present study indicates that SirT7 transcribes *fascin* to contribute to hyperglycemia-induced EndMT in DN patients.

## Introduction

Diabetic nephropathy (DN), which is the main cause of end-stage renal disease, is the most serious complication of diabetes [ 1, 2]. Once the disease progresses to end-stage renal disease, the mortality increases and the cost of treatment increases [ 3, 4]. Moreover, current treatments can delay the progression of DN, and effective treatment approaches are limited. Therefore, studies to explore the potential mechanisms of DN are urgently needed.

DN is characterized by impaired glomerular filtration capacity. The glomerular filtration barrier is constructed by human glomerular endothelial cells (HGECs), the glomerular basement membrane and podocytes. Damage to any part of the glomerular filtration barrier enhances glomerular permeability and leads to proteinuria [5]. Glomerular endothelial-to-mesenchymal transition (EndMT) was shown to play an important role in DN [6]. EndMT is defined as a reduction in the endothelial phenotype and an increase of mesenchymal phenotype [7]. EndMT in HGECs is considered the initial process of HGEC injury and is the origin of collagen-generating myofibroblasts contributing to fibrosis in DN [ 8, 9]. In addition, blocking EndMT relieves fibrosis and improves renal dysfunction in DN [10].

As a specific form of epithelial-to-mesenchymal transition, EndMT may involve common regulators of epithelial-to-mesenchymal transition [11]. Fascin was reported to play a crucial role in epithelial-to-mesenchymal transition [12] and renal fibrosis [13]. However, whether fascin participates in EndMT in DN is still unknown.

Epigenetic modifications play important roles in DN [14], and histone modifications play the most important role in DN [15]. Histone modification performs physiological functions by regulating downstream gene transcription. Our previous studies indicated that histone methylation participates in the occurrence and progression of DN via the modulation of alpha-enolase, perforin-2, protein tyrosine phosphatase 1B and phosphatase and tensin homologous transcription [ 16‒ 19]. Moreover, SirT7-mediated histone acetylation is involved in hyperglycemia-mediated endothelial inflammation via modulation of death-associated protein kinase 3 transcription [20]. However, whether SirT7 also participates in EndMT in DN is still not well known.

In the present study, we explored the underlying mechanism by which SirT7 participates in EndMT in DN. Our results indicated that SirT7 participates in EndMT in DN via modulation of *fascin* transcription.

## Materials and Methods

### Rat model

The present study complied with the Guidelines for the Care and Use of Laboratory Animals issued by the Committee on the Management and Use of Laboratory Animals of Fudan University Shanghai Cancer Center (license number: FUSCC-IACUC-S20210456). Male Sprague Dawley rats weighing 300‒400 g were used in the present study. The rats were raised under a 12/12-h light/dark cycle and in a temperature-controlled environment (22‒25°C). The animals underwent unilateral nephrectomy under anesthesia (isoflurane 3%–4% induction and 1.5%‒2.5% maintenance). After unilateral nephrectomy, the rats were raised for 9 weeks. The animals that received a single intraperitoneal injection of citrate buffer (0.1 M, pH 4.5) three weeks after unilateral nephrectomy were defined as the control group (Con). Animals that received a high-sugar and high-fat diet after unilateral nephrectomy and an intraperitoneal injection of streptozotocin (STZ, 50 mg/kg) three weeks after unilateral nephrectomy were defined as the DN group. To determine the therapeutic effect of SirT7 against DN, control vector- or AAV-SirT7-treated animals were injected into the contralateral kidney at the time of unilateral nephrectomy.

### Immunohistochemistry (IHC)

Rat kidney tissue samples were paraffin-embedded, and IHC was subsequently performed using standard protocols. Briefly, the paraffin sections were incubated with primary antibodies at 4°C overnight. After incubation with secondary antibody at room temperature for 1.5 h, the paraffin sections were stained with a DAB Detection kit (GeneTech, Shanghai, China) and counterstained with haematoxylin. Finally, sections were examine under an optical microscope. Antibodies used in the present study are shown in Table 1.

**Table:** **Table 1** Information of antibodies used in this study

Antibody	Information
SirT7	Cell Signaling Technology, USA
Fascin1	ProteinTech, Wuhan, China
α-SMA	ProteinTech
CD31	ProteinTech
Vimentin	ProteinTech
HRP-anti-mouse	ProteinTech
HRP-anti-rabbit	ProteinTech

### Cell culture and treatment

HGECs were obtained from Procell (Wuhan, China) and cultured in DMEM supplemented with 10% fetal bovine serum (FBS) and 1% penicillin‒streptomycin solution (PS) at 37°C in a 5% CO _2_ atmosphere. HGECs were cultured in 25 mM glucose (high glucose) DMEM for 3 days to establish a cell model of DN. Mannitol was added to normal medium (5 mM) DMEM to achieve the same osmotic pressure as the high glucose medium to exclude the effect of osmotic pressure.

### shRNA and plasmid treatments

After the HGECs were inoculated and reached 70%‒80% confluence, they were transfected with the SirT7 overexpression plasmid (SirT7-OE), SirT7 shRNA or fascin1 shRNA using Lipofectamine 2000 (Invitrogen, Carlsbad, USA). The ratio of plasmid or shRNA to Lipofectamine 2000 reagent was 1 mg/1.2 mL. The sequences of shRNA used in this study are shown in Table 2.

**Table:** **Table 2** The sequences of shRNAs used in this study

Name	Sequence (5′→3′)
shRNA-SirT7-a	CCAAATACTTGGTCGTCTA
shRNA-SirT7-b	GAAAGGGAGAAGCGTTAGT
shRNA-fascin1-a	GCCTGAAGAAGAAGCAGATCT
shRNA-fascin1-b	GCTGGTCGCTGCAGTCCGAGG
shRNA-fascin1-c	GCAAGTTTGTGACCTCCAAGA
shRNA-control	CAACAAGATGAAGAGCACCAA

### qPCR analysis

Total RNA was extracted using an EZ-press RNA Purification kit (EZBioscience, Roswell, USA). Hifair® II 1st Strand cDNA Synthesis SuperMix (Yeasen, Shanghai, China) was used to synthesize cDNA for qPCR. Then, qPCR was performed with Hieff UNICON® qPCR TaqMan Probe Master Mix (Yeasen) on an ABI7500 Real-Time PCR system (Applied Biosystems, Foster City, USA). The sequences of the qPCR primers used in this study are shown in Table 3.

**Table:** **Table 3** Sequences of primers used for the real-time RT-PCR analysis

Gene	Sequence (5′→3′)
*SirT7*	F: TGGAGTGTGGACACTGCTTCAG R: CCGTCACAGTTCTGAGACACCA
*Fascin1*	F: GCTGCTACTTTGACATCGAGTGG R: CTTCTTGGAGGTCACAAACTTGC
*α-SMA*	F: CCACCCCGCAGTCACTTTC R: ATGTATGTACACGTTATAAACACTGTG
*CD31*	F: AAGTGGAGTCCAGCCGCATATC R: ATGGAGCAGGACAGGTTCAGTC
*Vimentin*	F: AGGCAAAGCAGGAGTCCACTGA R: ATCTGGCGTTCCAGGGACTCAT

### Hematoxylin and eosin (HE) staining

The paraffin sections were placed in an oven at 60°C for 1‒2 h and dewaxed with xylene (National Pharmaceutical Group, Beijing, China) and ethanol. Hematoxylin (Sigma-Aldrich, St Louis, USA) was used to stain the nuclei for about 10 min and eosin (Sigma-Aldrich) was used to stain the cytoplasm for 30 s. Finally, the sections were sealed with neutral balsam (National Pharmaceutical Group), dried at room temperature, and observed under an optical microscope (Nikon, Tokyo, Japan).

### Masson trichrome staining

After paraffin sections were dewaxed, weigert iron hematoxylin (1:1 mixture of liquid A and liquid B) (National Pharmaceutical Group) was first stained for 10 min, rinsed with running water, and differentiated by 1% hydrochloric acid alcohol (National Pharmaceutical Group). Then the tissues were stained with acid fuhong-ponceau solution (National Pharmaceutical Group) for about 8 min. After washing again, phosphomolybdate solution (OKA Biotechnology, Beijing, China) was used to differentiate and stain the sections for 3‒5 min. Until the tissues were observed under the microscope with varying degrees of red, they were dyed with aniline blue solution (National Pharmaceutical Group) for 5 min. After the last washing, dehydrated with anhydrous alcohol, transparent with xylene, sections were sealed for microscopic examination.

### Western blot analysis

Whole-cell extracts from different groups of HGECs were prepared using cell lysis buffer (Cell Signaling Technology, Danvers, USA). The protein samples (50 μg) were boiled in loading buffer at 100°C for 10 min, separated by 8%‒10% SDS-PAGE, and transferred to PVDF membranes. The membranes were blocked with protein-free rapid blocking buffer (Beyotime Biotechnology, Shanghai, China) for 1 h, after which all the membranes were incubated with specific primary antibodies at 4°C overnight. After washing 5 times, the membranes were incubated with secondary antibodies at room temperature for 1 h. The membranes were subsequently washed with PBST for 5 additional times. An ECL system (Beyotime Biotechnology) was used to detect the protein signals. The mean densities of the protein bands were analyzed using ImageJ.

### Chromatin immunoprecipitation (ChIP) assay

ChIP assays were performed with a Simple ChIP kit (17-371RE; EMD Millipore, Billerica,USA) according to the manufacturer’s directions. Briefly, the cells (1×10 ^7^) were fixed with 1% formaldehyde for 10 min at room temperature to crosslink the DNA and the proteins. The cross-linking reaction was then stopped with the use of 2.5 mM glycine. Chromatin was sheared with the use of ultrasound. After centrifugation, the supernatant was incubated with specific primary antibodies or IgG at 4°C overnight. Agarose beads (17-371RE; EMD Millipore) were applied to immunoprecipitate the proteins. The mixture was incubated at 65°C for 4‒5 h to reverse cross-linking DNA with proteins. Finally, the DNA was purified by centrifugation and verified by electrophoresis. The oligonucleotide sequences of primers used for *fascin1* are listed in Table 4.

**Table:** **Table 4** Sequences of primers used for *fascin1* promoters

Gene	Sequence (5′→3′)
Fascin1-ChIP1(‒1764~‒1885)	F: CTCACATCTGTACCCAATCTAGAGC R: AATAGACGATAGAAAATGCCTTGG
Fascin1-ChIP2(‒1417~‒1556)	F: GGAATCCTCTTTCCTCAGCCTC R: CTCAACCGCAAGCCAACATG
Fascin1-ChIP3(‒433~‒586)	F: GCCTCAAGGAACCACATCTCTG R: GAGGCAGACGAGGGAAAGAGG
Fascin1-ChIP4(‒288~‒396)	F: CCTCCAGGCAGCCCTCAGA R: CCTCGCTAGGAGCAAGGACGA

### Statistical analysis

Data are shown as the mean±standard deviation. The comparison of the means of two groups was conducted by two-tailed unpaired *t* tests. One-way ANOVA followed by Bonferroni-corrected pairwise comparison was employed to compare the means of more than 2 groups. *P*<0.05 was considered statistically significant.

## Results

### Occurrence of EndMT and augmentation of fascin level *in vivo*

**Figure 1:**
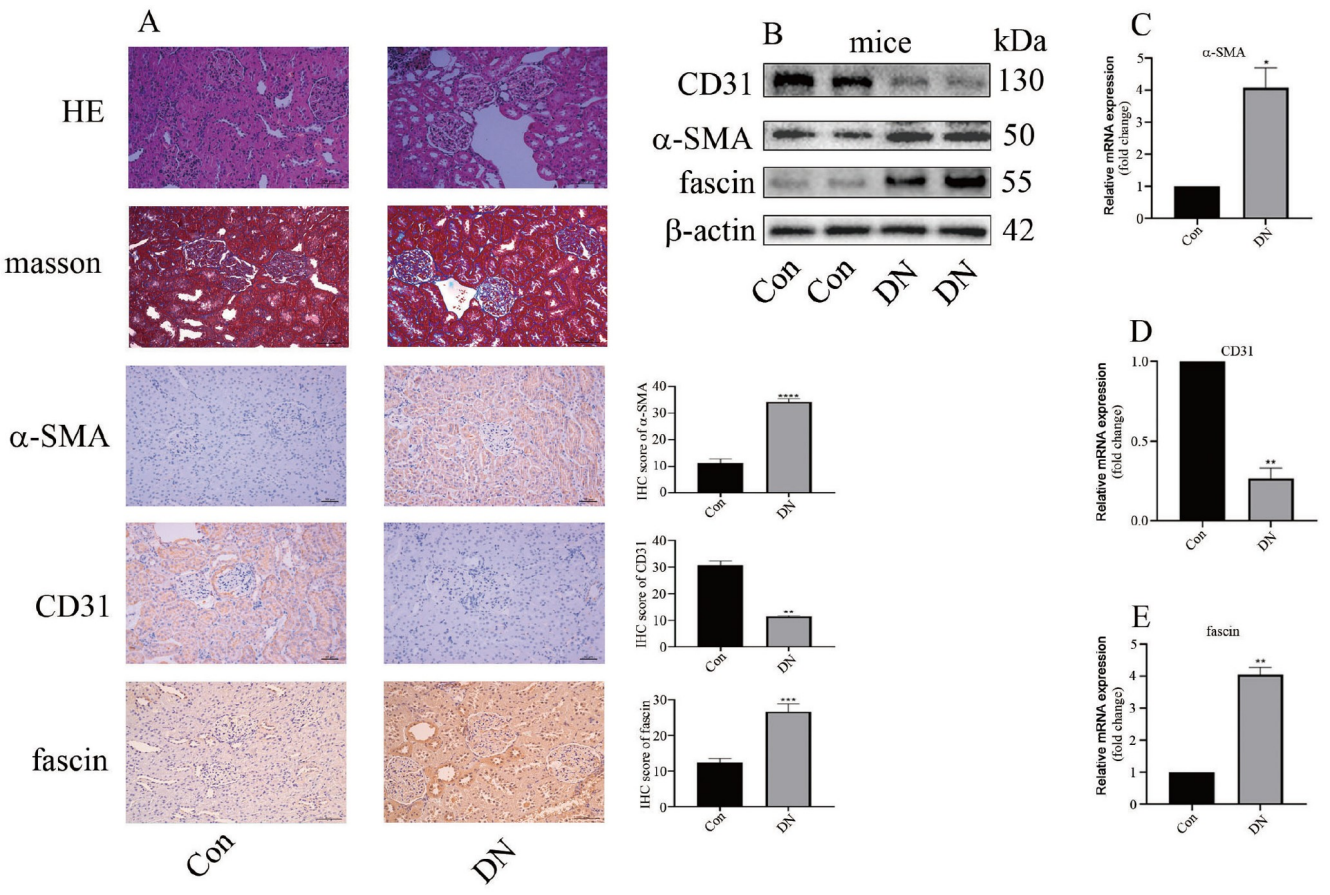
Figure 1 EndMT and fascin levels in control and DN rats (A) HE staining, Masson staining, and IHC staining of α-SMA, CD31 and fascin in the kidneys of DN animals. (B) Western blot analysis results showing the expressions of α-SMA, CD31 and fascin in the kidneys of DN animals. (C) qPCR results indicated that the mRNA level of α-SMA was increased in the kidneys of DN rats. (D) qPCR results indicated that the mRNA level of CD31 increased in the kidneys of DN rats. (E) qPCR results indicating that the mRNA level of fascin increased in the kidneys of DN rats (*P<0.05 vs Con; **P<0.01 vs Con, ***P<0.001 vs Con, ****P<0.0001 vs Con; n=5).

The characteristics of the rats in this study are shown in Table 5. Hematoxylin and eosin (HE) staining and Masson trichrome staining revealed renal damage and interstitial fibrosis in the glomeruli of DN rats ( Figure 1A). Moreover, IHC staining of renal biopsy specimens from DN rats indicated that the expression of α-smooth muscle actin (α-SMA) was increased, while the expression of CD31 was decreased ( Figure 1A). Fascin has been reported to play an important role in renal fibrosis [13]; therefore, we examined the level of fascin in the renal biopsy specimens of DN animals. IHC staining revealed that fascin expression was increased in the kidneys of DN animals ( Figure 1A). Consistently, western blot analysis and qPCR results indicated that the levels of α-SMA and fascin were increased, while the level of CD31 was decreased in DN rats ( Figure 1B‒E). Our results demonstrated that fascin may regulate EndMT in DN.

**Table:** **Table 5** Characteristics of rats in control (Con), diabetic nephropathy (DN), DN with empty vector (SirT7+AVV), and DN with SirT7 overexpression (DN+AVV-SirT7) groups

Rat variables	Con	DN	DN+AVV	DN+AVV-SirT7
Weight (g)	372.6±39.5	530.0±68.9***	538.9±30.4	414.8±40.0 ^###^
Weight of kidney (g)	1.65±0.15	2.89±0.68**	2.92±0.57	2.28±0.30 ^#^
FBS (mM)	4.65±1.19	10.33±2.51***	10.1±1.40	5.46±1.38 ^###^
HbA1c (%)	4.87±1.05	11.92±1.54 ^***^	11.7±1.95	6.23±1.65 ^###^
TG (mM)	0.961±0.25	4.36±1.46 ^**^	4.14±1.32	3.23±1.15
UA (μM)	232.5 (196.8, 256.2)	316.9 (312.3, 365.8) ^***^	319.1 (289.1,386.6)	280.8 (279.7,284.4) ^#^
Scr (μM)	50.39±4.28	52.78±7.17	54.4±10.1	51.0±8.36
BUN (mM)	6.88±0.98	9.32±0.70 ^***^	10.0±1.29	8.16±1.14 ^##^
TC (mM	1.30 (1.25, 1.45)	3.10 (2.69, 3.88) ^***^	3.11 (2.73,3.32)	2.12 (1.72,2.46) ^##^
LDL (mM)	0.485 (0.460, 0.555)	1.195 (0.945,1.320) ^***^	1.06 (0.81,1.15)	0.700 (0.61,0.72) ^###^
HDL (mM)	0.625±0.58	1.023±0.21 ^**^	1.04±0.16	0.79±0.96 ^##^

Data are expressed as the mean±SD and compared using an independent sample T test. Data that are not normally distributed were expressed as median (IQR) and compared using the MannWhitney U test. *Compared to the control (Con) group; ^#^Compared to the DN with SirT7 empty vector (SirT7+AVV) group. * *P*<0.05, ** *P*<0.01, *** *P*<0.001; *n*=8 per group. FBS, fasting blood sugar; HbA1c, glycosylated hemoglobin; TG, triglyceride; UA, uric acid; Scr, serum creatinine; BUN, blood urea nitrogen; TC, total cholesterol; LDL, low density lipoprotein; HDL, high density lipoprotein.

### High glucose induces EndMT in hyperglycemic HGECs via upregulation of fascin levels

**Figure 2:**
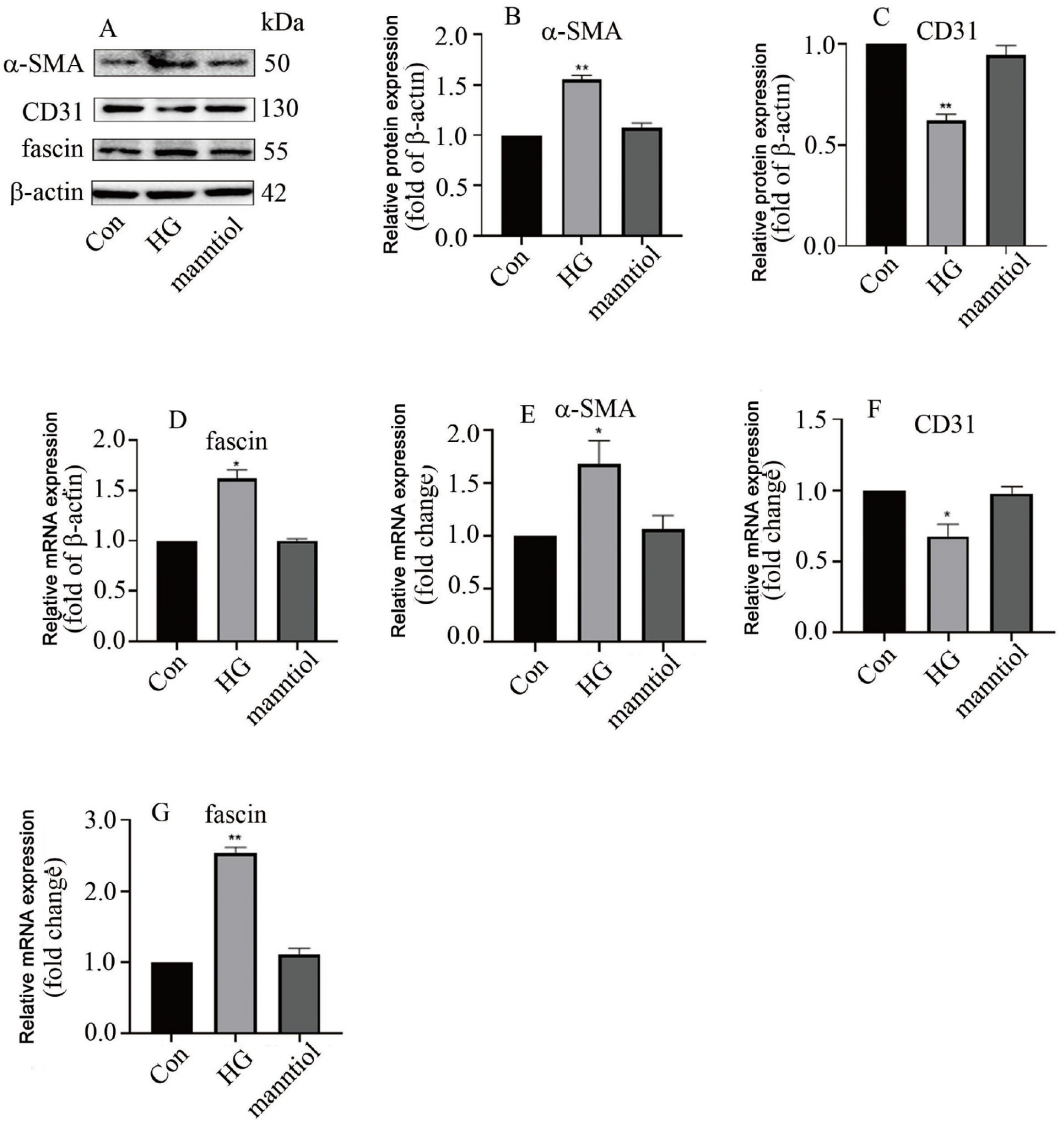
Figure 2 High glucose concentration upregulated fascin expression and induces EndMT in HGECs (A) Western blot analysis results showed that high glucose treatment increased α-SMA and fascin levels and decreased CD31 expression. (B) Quantification of the α-SMA band density. (C) Quantification of the CD31 band density. (D) Quantification of fascin band density. (E) qPCR analysis showed that high glucose treatment enhanced α-SMA expression. (F) qPCR analysis indicated that high glucose treatment reduced CD31 level. (G) qPCR analysis showed that high glucose treatment augmented fascin expression (*P<0.05 vs Con; **P<0.01 vs Con; n=5).

**Figure 3:**
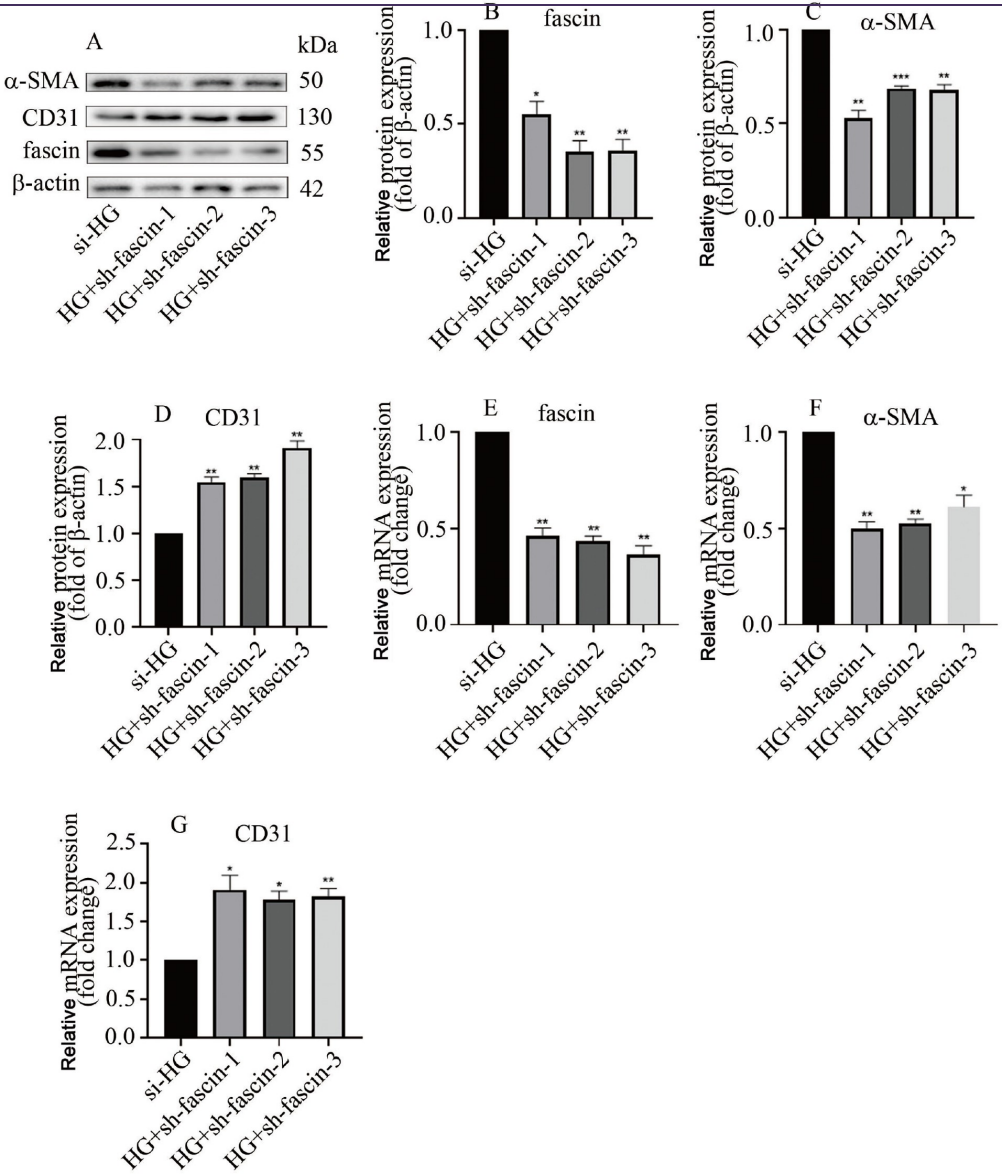
Figure 3 *Fascin* silencing inhibited EndMT in hyperglycaemic HGECs (A) Western blot analysis indicated that fascin silencing decreased α-SMA level and increased CD31 level in hyperglycemic HGECs. (B) Quantification of the α-SMA band density. (C) Quantification of the CD31 band density. (D) Quantification of fascin band density. (E) The effectiveness of the combinations was verified via qPCR analysis. (F) qPCR analysis showed that fascin silencing decreased α-SMA level in hyperglycaemic HGECs. (G) qPCR analysis showed that fascin silencing increased CD31 expression in hyperglycaemic HGECs (*P<0.05; **P<0.01, ***P<0.001; n=5).

To further determine whether fascin participates in EndMT in DN, we constructed a cell model in this study with the use of HGECs. Our data indicated that high glucose treatment increased α-SMA and fascin expressions but decreased CD31 expression at both the protein ( Figure 2A‒D) and mRNA levels ( Figure 2E‒G). These data were quite similar to those obtained for DN rats. Next, we downregulated fascin expression in hyperglycaemic HGECs, and the effect of sh-fascin was confirmed via western blot analysis ( Figure 3A) and qPCR ( Figure 3B). Our results indicated that inhibition of fascin expression increased CD31 expression but decreased α-SMA level in hyperglycemic HGECs ( Figure 3). Our data revealed that high glucose induces EndMT via an increase in fascin level in HGECs.

### SirT7 expression is reduced in DN animals and hyperglycemic HGECs

**Figure 4:**
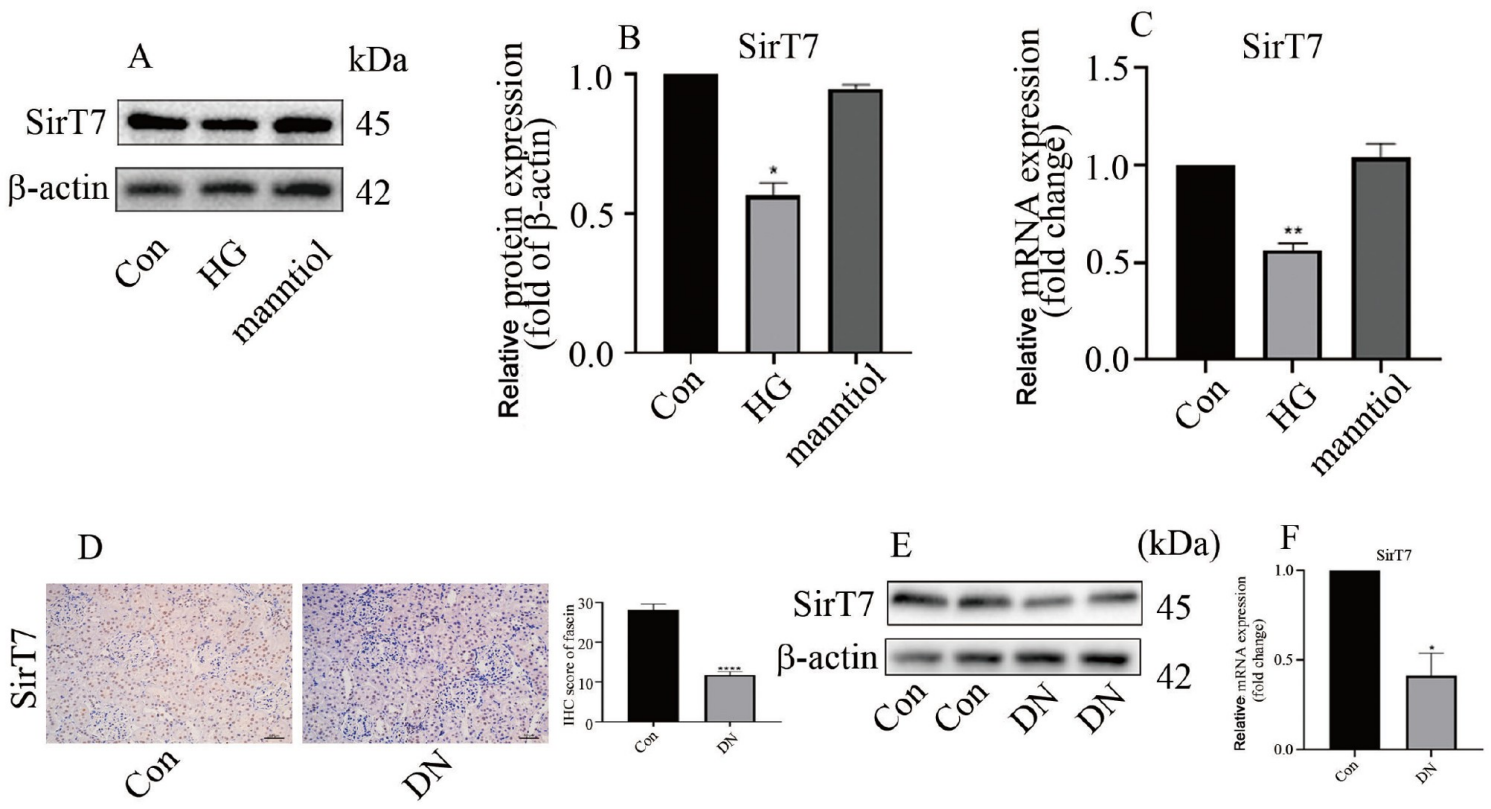
Figure 4 SirT7 level was reduced in hyperglycemic HGECs and in the kidneys of DN rats (A) Western blot analysis results indicated that SirT7 expression was decreased in hyperglycaemic HGECs. (B) Quantification of SirT7 band density. (C) qPCR analsyis indicated that SirT7 expression was decreased in hyperglycaemic HGECs. (D) Immunostaining data indicating that SirT7 expression was decreased in the kidneys of DN rats. (E) Western blot analysis results indicating that SirT7 expression was decreased in the kidneys of DN rats. (F) qPCR results indicating that SirT7 expression was decreased in the kidneys of DN rats (*P<0.05, **P<0.01, ****P<0.0001; n=5).

Histone modification reportedly plays an important role in DN [ 14, 15]. Our previous studies demonstrated that histone methylation participates in the occurrence and progression of DN [ 16‒ 19]. Moreover, SirT7-mediated histone acetylation participates in hyperglycemia-mediated endothelial inflammation [20]. However, whether SirT7 participates in EndMT in DN is still unknown. The present study demonstrated that high glucose treatment decreased SirT7 protein ( Figure 4A,B) and mRNA ( Figure 4C) levels in HGECs. Consistently, SirT7 expression was also inhibited in the kidneys of DN animals ( Figure 4D‒F). These data indicated that the level of SirT7 decreased in DN rats and hyperglycemic HGECs and SirT7 may participate in EndMT in DN patients.

### SirT7 participates in EndMT in hyperglycaemic HGECs via modulation of *fascin* transcription

**Figure 5:**
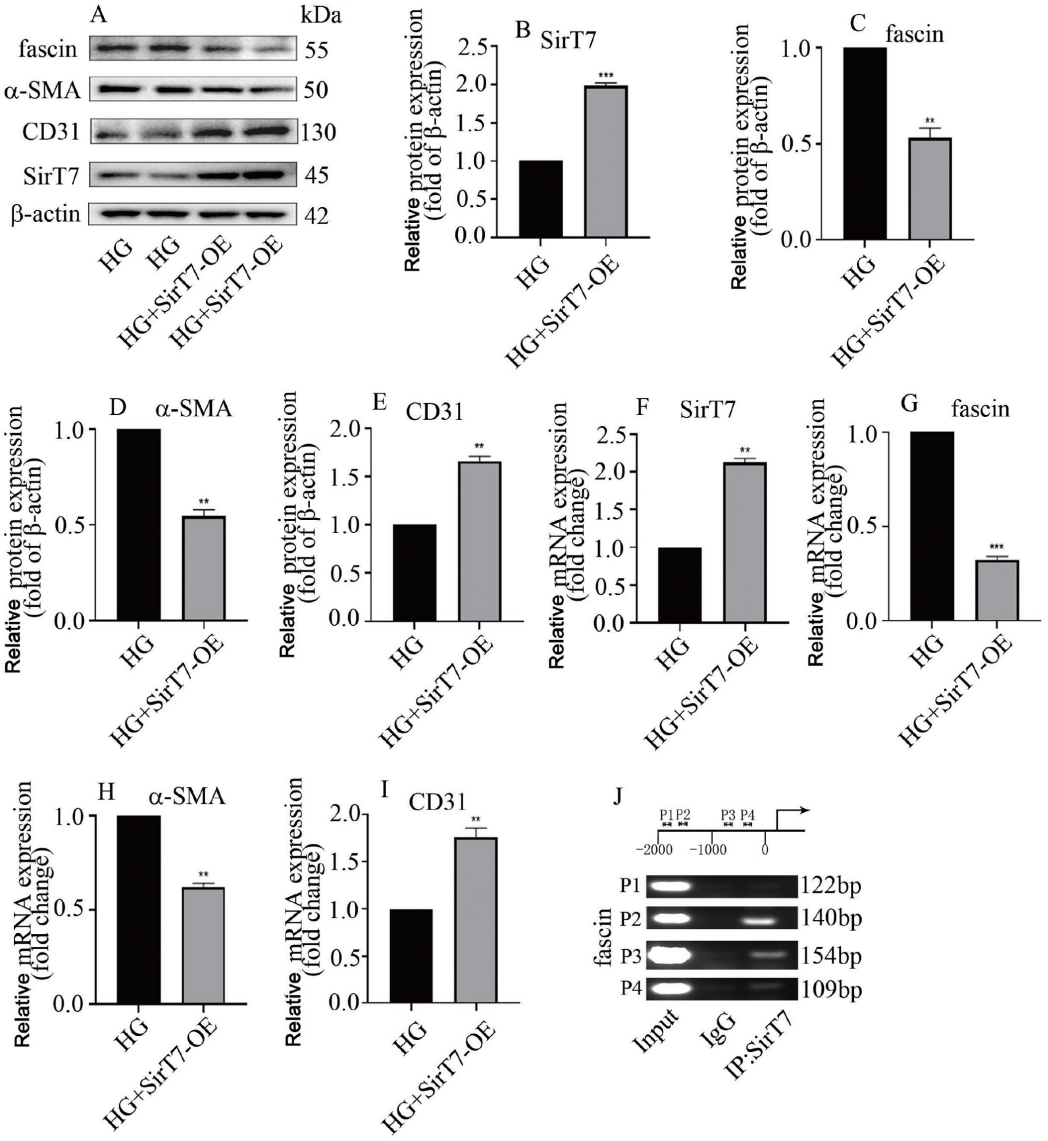
Figure 5 SirT7 overexpression decreased fascin expression and inhibited EndMT in hyperglycemic HGECs (A) Western blot analysis results indicated that SirT7 overexpression inhibited fascin and α-SMA levels, and increased CD31 expression in hyperglycaemic HGECs. (B) Quantification of SirT7 band density. (C) Quantification of fascin band density. (D) Quantification of the α-SMA band density. (E) Quantification of the CD31 band density. (F) The effectiveness of SirT7 overexpression was confirmed via qPCR analysis. (G) qPCR analysis indicated that SirT7 overexpression decreased fascin level in hyperglycaemic HGECs. (H) qPCR analysis was used to determine whether SirT7 overexpression decreased α-SMA level in hyperglycaemic HGECs. (I) qPCR analysis showed that SirT7 overexpression enhanced CD31 level in hyperglycaemic HGECs. (J) ChIP assay showed that SirT7 binds to the promoter of fascin (*P<0.05, **P<0.01, ***P<0.001; n=5).

**Figure 6:**
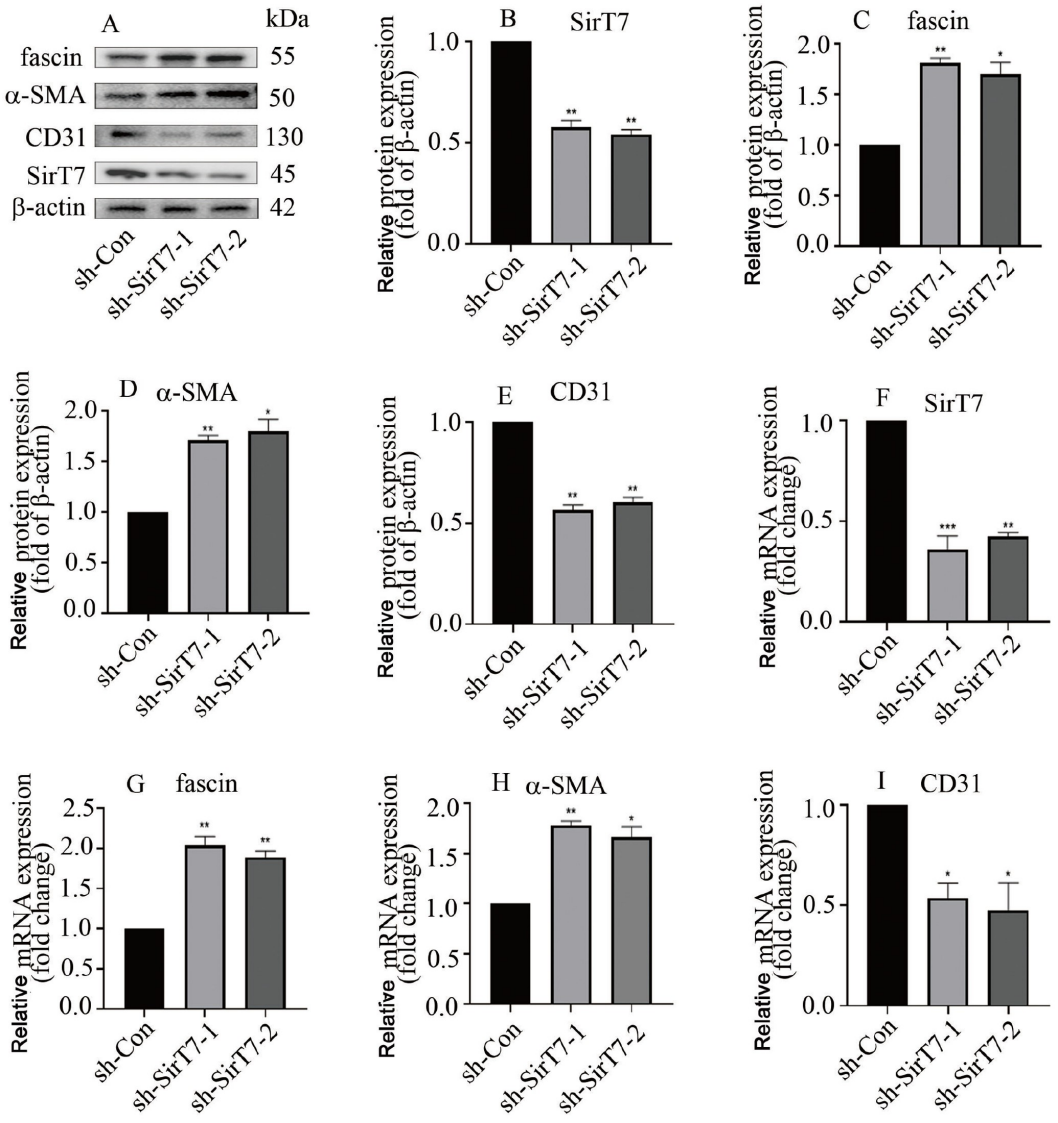
Figure 6 *SirT7* silencing increased fascin expression and induced EndMT in HGECs (A) Western blot analysis indicated that SirT7 silencing increased fascin and α-SMA levels and decreased CD31 expression in HGECs. (B) Quantification of SirT7 band density. (C) Quantification of fascin band density. (D) Quantification of the α-SMA band density. (E) Quantification of the CD31 band density. (F) The effectiveness of SirT7 silencing was confirmed via qPCR. (G) qPCR analysis indicated that SirT7 silencing increased fascin expression in HGECs. (H) qPCR analysis showed that SirT7 silencing enhanced α-SMA expression in HGECs. (I) qPCR analysis showed that SirT7 silencing reduced CD31 expression in HGECs (*P<0.05, **P<0.01, ***P<0.001; n=5).

To determine whether SirT7 modulates EndMT via modulation of *fascin* transcription, both loss-of-function and gain-of-function approaches were used in this study. Our data indicated that SirT7 overexpression decreased α-SMA and fascin levels but increased CD31 expression at the protein ( Figure 5A‒E) and mRNA ( Figure 5F‒I) levels. Moreover, ChIP assay revealed that SirT7 bound to the promoter of *fascin* ( Figure 5J). Furthermore, *SirT7* silencing decreased CD31 expression and increased fascin and α-SMA protein ( Figure 6A‒E) and mRNA ( Figure 6F‒I) levels. These data demonstrated that SirT7 participates in EndMT in hyperglycaemic HGECs via modulation of *fascin* transcription.

### SirT7 overexpression inhibits EndMT and improves renal dysfunction in DN animals

**Figure 7:**
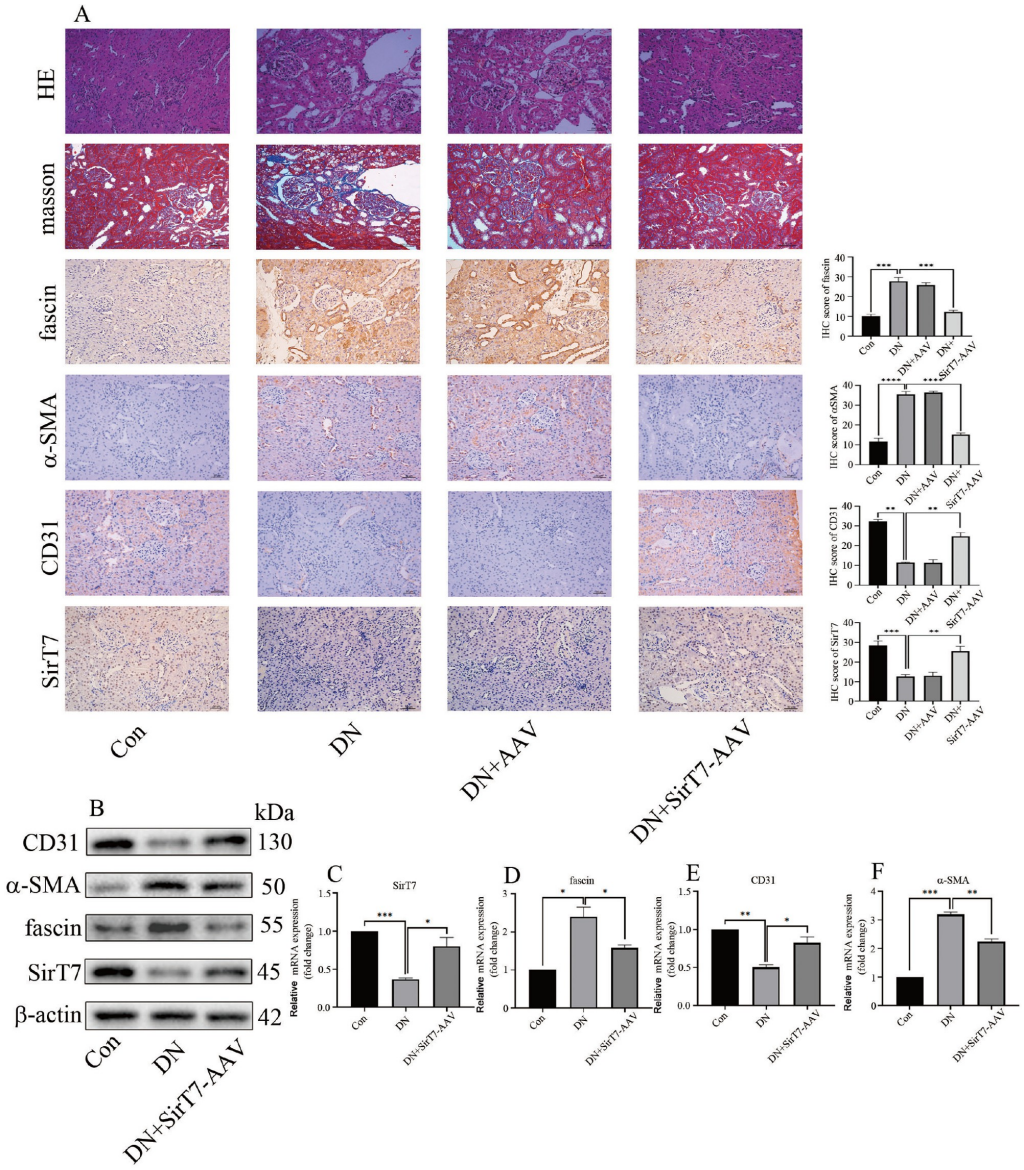
Figure 7 Overexpression of SirT7 inhibited EndMT and improved renal dysfunction in DN rats (A) HE staining, Masson staining, and IHC results for α-SMA, CD31, fascin and SirT7 expressions in the kidneys of the rats after the corresponding treatment. (B) Western blot analysis results showing the expressions of α-SMA, CD31, fascin and SirT7 in the kidneys of the rats after the corresponding treatment. (C) qPCR results showing SirT7 expression in the kidneys of the rats after the corresponding treatment. (D) qPCR results of fascin in the kidneys of the rats with the corresponding treatment. (E) qPCR results showing CD31 expression in the kidneys of the rats with the corresponding treatment. (F) qPCR analysis of α-SMA expression in the kidneys of the rats with the corresponding treatment (*P<0.05, **P<0.01, ***P<0.001, ****P<0.0001; n=5).

**Figure 8:**
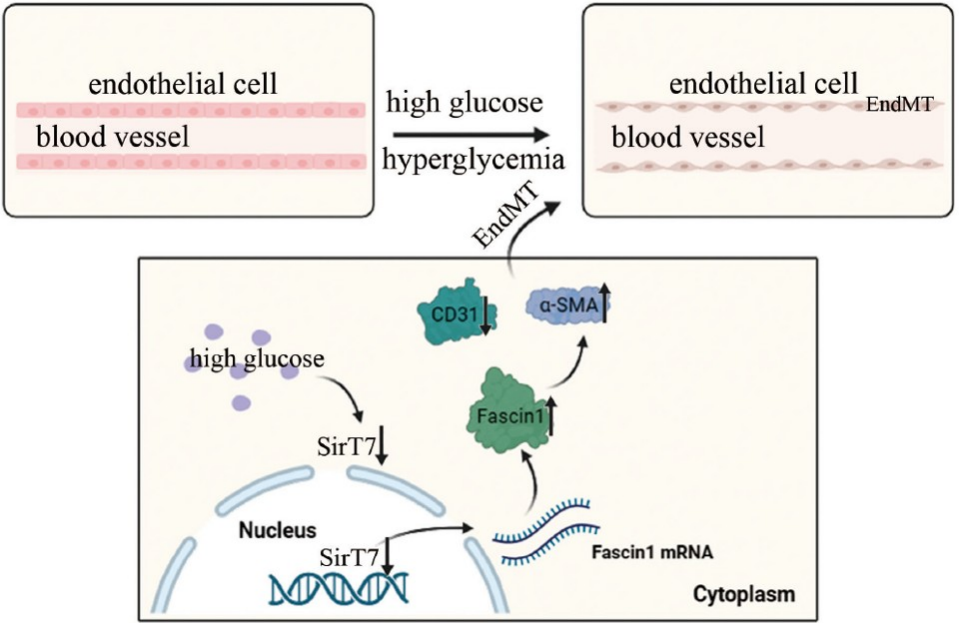
Figure 8 The mechanism diagram of SirT7-mediated transcription of fascin contributes to EndMT in diabetic nephropathy. The expression of SirT7, which inhibits the transcription of fascin1, is down-regulated in hyperglycemic HGECs and the kidneys of DN rats. Fascin1 with increased expression in hyperglycemia further induces EMT.

To determine the inhibitory effect of SirT7 overexpression on EndMT *in vivo*, we used AAV-SirT7 in this study. The effectiveness of AAV-SirT7 is shown in Figure 7A‒C. Our results demonstrated that Sirt7 upregulation reduced renal injury and fibrosis ( Figure 7A). Moreover, IHC assay revealed that SirT7 overexpression reduced fascin ( Figure 7C) and α-SMA levels but increased CD31 expression in the kidneys of DN rats ( Figure 7A). Consistently, Western blot analysis and qPCR results indicated that an increase in SirT7 expression decreased fascin expression and inhibited EndMT in DN rats ( Figure 7B‒F). In addition, SirT7 upregulation improved renal dysfunction in DN animals ( Table 5). These results suggested that SirT7 augments the inhibition of EndMT in DN rats, thus improving renal dysfunction. The mechanism diagram of SirT7-mediated transcription of fascin contributes to EndMT in diabetic nephropathy is shown in Figure 8.

## Discussion

The core findings of the present study were that hyperglycemia is involved in the modulation of EndMT via an increase in fascin expression, thus contributing to the occurrence and progression of DN. Moreover, SirT7 was decreased in hyperglycemic HGECs and in the kidneys of DN rats. Mechanistic studies indicated that SirT7 regulates fascin transcription to induce EndMT in hyperglycemic HGECs.

The epithelial-to-mesenchymal transition, which involves intricate cell phenotypic reconstruction, plays an important role in tissue and organ damage [21]. Previous studies have demonstrated that epithelial-to-mesenchymal transition (EMT) of renal epithelial cells plays an important role in kidney fibrosis [22]. Recently, glomerular EndMT was indicated to be involved in DN [ 5, 6]. The present study revealed that CD31 expression was reduced and that α-SMA expression was augmented in the kidneys of DN rats and hyperglycemic HGECs. The present study was quite similar to a recent study which indicated that EndMT plays a crucial role in DN [23]. It was deduced that epithelial-to-mesenchymal transition and the EndMT may be associated with other factors [11]. Fascin acts as an actin-binding protein that is enriched in the actin bundles of spikes and filopodia [ 24, 25]. Moreover, fascin is involved in filopodia construction to increase cell migration [26]. In addition, fascin promotes epithelial-to-mesenchymal transition [ 27, 28]. In the present study, fascin was found to be augmented in the kidneys of DN rats and hyperglycemic HGECs. Additionally, *fascin* silencing enhanced CD31 level and reduced α-SMA level, thus suppressing EndMT in hyperglycemic HGECs. These data indicated that fascin plays a crucial role in the modulation of EndMT in DN patients.

Epigenetic modifications have been found to play an important role in DN [14], and histone modifications play the most important role in DN [15]. Our previous studies indicated that lysine methyltransferase 5A-mediated histone methylation regulates enolase 1 [16] and perforin-2 [17], thus playing a crucial role in EndMT in DN. Moreover, SET domain containing lysine methyltransferase 8-mediated histone methylation modulates protein tyrosine phosphatase 1B [18] and phosphatase and tensin homolog [26] transcription to induce endothelial inflammation in diabetes. Furthermore, our study indicated that SirT7-mediated histone acetylation participates in endothelial inflammation via modulation of death-associated protein kinase 3 expression [20]. In the present study, we found that SirT7 overexpression inhibited α-SMA expression but elevated CD31 expression in hyperglycaemic HGECs. Moreover, silencing of *SirT7* upregulated α-SMA expression and decreased CD31 expression. Furthermore, AAV-SirT7 increased CD31 expression, decreased α-SMA level, and improved renal function in DN rats. These data indicated that SirT7 is involved in the regulation of EndMT in DN patients. In addition, SirT7 bound to the promoter of *fascin*, which indicated that SirT7 modulates EndMT via the regulation of fascin transcription. Our research and that of other scholars indicated that SirT7 participates in endothelial inflammation [20], Podulus apoptosis [29], and EndMT in DN. Therefore, SirT7 plays an important role in DN.

Nevertheless, this study has several limitations. First, HGECs were used to construct a cellular model, and other primary endothelial cells should be used to confirm the results of the present study. Second, the potential mechanism by which fascin induces EndMT in hyperglycaemic HGECs is still not well known and deserves further research.

In conclusion, this study demonstrated that SirT7 expression decreased, fascin expression increased, and that EndMT occurred in DN rats. In addition, this study indicated that high concentration of glucose induces EndMT via an increase in fascin level in hyperglycemic HGECs. Moreover, SirT7 was found to negatively regulate *fascin* transcription to participate in the modulation of EndMT in DN. However, upregulation of SirT7 expression decreased *fascin* transcription, thus inhibiting EndMT and improving renal function in hyperglycemic HGECs and DN mice. Our study revealed that SirT7 may be an underlying therapeutic target for DN.
